# Perinatal outcome in fresh versus frozen embryo transfer in ART cycles

**Published:** 2016-03

**Authors:** Ali Aflatoonian, Mohammad Ali Karimzadeh Maybodi, Nastaran Aflatoonian, Nasim Tabibnejad, Mohammad Hossein Amir-Arjmand, Mehrdad Soleimani, Behrouz Aflatoonian, Abbas Aflatoonian

**Affiliations:** 1 *Madar Hospital, Yazd, Iran.*; 2 *Infertility Research Center, Shahid Sadoughi University of Medical Sciences, Yazd, Iran.*; 3 *Stem Cell Biology Research Center, Shahid Sadoughi University of Medical Sciences, Yazd, Iran.*

**Keywords:** *Frozen-thawed embryo transfer*, *Fresh embryo transfer*, *Perinatal outcome*, *Pregnancy outcome*

## Abstract

**Background:**

Despite of higher pregnancy rate after frozen embryo transfer (FET) which is accepted by the majority of researches, the safety of this method and its effect on neonatal outcome is still under debate.

**Objective:**

The aim of this study was to evaluate pregnancy and neonatal outcome of FET compare to fresh cycles.

**Materials and Methods:**

In this study,1134 patients using fresh ET and 285 women underwent FET were investigated regarding live birth as primary outcome and gestational age, birth weight, gender, multiple status, ectopic pregnancy, still birth and pregnancy loss as secondary outcomes.

**Results:**

Our results showed that there is no difference between FET and fresh cycles regarding live birth (65.6% vs. 70.4% respectively). Ectopic pregnancy, still birth and abortion were similar in both groups. The mean gestational age was significantly lower among singletons in FET group compared to fresh cycles (p=0.047). Prematurity was significantly elevated among singleton infants in FET group (19.6%) in comparison to neonates born after fresh ET (12.8%) (p=0.037).

**Conclusion:**

It seems that there is no major difference regarding perinatal outcome between fresh and frozen embryo transfer. Although, live birth is slightly increased in fresh cycles and prematurity was significantly increased among singleton infants in FET group.

## Introduction

Introduction of embryo cryopreservation was a revolution in assisted reproductive technology (ART). Transfer of frozen embryos has been increasingly used during the past few decades, as it is a well-known part of ART nowadays. Frozen embryo transfer (FET) has several advantages and among them, the similar or even higher pregnancy and live birth rate compare to fresh cycles is of great importance (1). In addition, higher pregnancy rate after FET compared to fresh cycles was reported in some randomized trials and meta-analysis (1-3). The other benefit of FET is possibility of embryo transfer in a natural, non-stimulated cycle. It is shown that an artificial cycle may adversely affect endometrial receptivity leading to implantation failure (2-6). 

FET also is a useful method for preserving extra good quality embryos in women with good response to ovarian stimulation, increasing elective single embryo transfer and avoiding multi-gestations as well as ovarian hyperstimulation syndrome. Regardless of cryopreservation technique improvements, safety aspects and its impact on the health of children born are uncertain. 

Overall, it is showed that around 26% of newborns after ART were premature with a lower mean gestational age and 3.5 times higher risk of rising prematurity (7). It is reported in some investigations that embryo freezing has not a negative impact on perinatal outcome in terms of low birth weight (LBW), preterm labour and small for gestational age (SGA) (8-10). However, Wennerholm *et al* showed a higher prenatal mortality rate among singletons born after FET (10). 

In addition, FET increases the risk of being born large for gestational age (LGA) (10, 11). In terms of the risk for major congenital anomalies among children born after FET, there is no significant increase compared with children born after fresh embryo transfer (8, 12). One study reported a higher major malformation rate in the children born after FET following intra cytoplasmic sperm injection (ICSI) (13). This study is a continuation of our previous one and evaluates neonatal outcomes after FET compared with fresh embryo transfer cycles (14). In the former study, we found similar neonatal outcomes regarding prematurity, LBW, stillbirth, neonatal death and major congenital anomalies between FET and fresh ET groups in two infertility clinics.

## Materials and methods

**Study population**

To study the impact of FET on neonatal outcome, in a cohort study, 300 women using FET and 1150 women undergoing fresh embryo transfer were compared. Participants remained in the cohort until the livebirth occurred. The study was conducted at Yazd Madar Hospital over a 4-years period between December 2010 and December 2014. This study was approved by the Ethics Committee of Research and Clinical Center for Infertility, Yazd University of Medical Sciences.

**Data Collection**

Data were collected from the hospital records. In addition a telephone questionnaire consists of data on maternal and neonatal factors was administered by a trained nurse based on patients and their husbands’ information. Patient’s data included maternal age at birth, duration and causes of infertility. The primary outcome was live birth. The secondary outcome variables were perinatal data contains gestational age, birth weight, gender, twin status, stillbirth, ectopic pregnancy (EP) and spontaneous abortion.

Outcomes were defined as followed: Preterm birth: <37 wks of gestational age at delivery. Small for gestational age (SGA): Birth weight less than 10^th^ centile for gestational age. LBW: <2500 gr at birth. Stillbirth: Fetal death more than 20 wks of gestational age. EP: Finding of extra uterine pregnancy by laparoscopy or ultrasound. Spontaneous abortion: Loss of pregnancy before 20 wks of gestation. The increased miscarriage rate in this survey is thought to be due to pregnancy loss calculation, according to chemical pregnancy in women with an initial positive β-hCG test.

**Ovarian stimulation protocol**

Two protocols were used for ovarian stimulation: GnRH agonist long protocol and GnRH antagonist protocol as described previously (15, 16).

**Embryo cryopreservation techniques and transfer protocols**

All embryos were morphologically evaluated on the second day after oocyte retrieval. Number of blastomeres and cytoplasmic fragmentation was assessed. Two embryos with good or excellent quality were transferred in fresh cycles, only in women over the age of 40 and regarding patients’ request, 3 embryos were transferred. All the extra embryos with less than 30% fragmentation were cryopreserved by vitrification method. 

During two step loading protocols, embryos were loaded with equilibration solution containing 7.5% dimethyl sulfoxide (DMSO) (Sigma-Aldrich) and 7.5% ethylene glycol (EG) (Sigma-Aldrich, Steinheim, Germany) in Ham’s F-10 media supplemented with 20% patient serum for 5-15 min at room temperature. Once the primary shrinkage and recovery, embryos were aspirated and placed into the vitrification solution 15% EG, 15% DMSO and 0.5 M sucrose (Merck, Darmstadt, Germany) in Ham’s F-10 medium supplemented with 20% patient serum for 50-60 sec at room temperature. The embryos then were loaded by a thin glass capillary tube into the cryotop and the samples were immediately submerged into liquid nitrogen for at least 2 months. 

For thawing, cryoprotectants were removed step by step using embryo thawing media (Vitrolife, Goteborg, Sweden) by insertion the Cryotop in thawing solution (1 M sucrose) for 50-60 sec and next into dilution solution (0.5 M sucrose) for 3 min, after that embryos placed in another dilution solution of 0.25 M sucrose for 3 min, all at room temperature. The thawed embryos were placed 4-5 times into washing solution (Ham’s F-10 +20% serum) before incubation. After embryo transferred to culture medium, they considered morphologically survived by 50% or more intact blastomeres and no injury to zonapllucida. Only intact or partly damaged embryos were transferred.

Endometrial preparation was performed using oral Estradiol Valerat (Estradiol Valerate, Aburaihan CO, Tehran, Iran) at the dose of 6 mg per day from the second day of menstrual cycle until the endometrial thickness reached more than 8 mm, and then 100 mg progesterone in oil (Progesterone, Aburaihan CO, Tehran, Iran) was injected or Cyclogest 400 mg (Collins & Co. Ltd, UK) was used daily. Estradiol and progesterone administered continuously until observation of fetal heart activity by ultrasound. Embryo transfer was done 3 days after the beginning of progesterone injection using a Labotect (Labotect, Gottingen, Germany) or Cook catheter (Laboratoire CCD, France).

**Statistical analysis**

The Statistical Package for the Social Science version 20 for windows (SPSS Inc, Chicago. IL, USA) was used for data analysis. Differences between normally distributed continuous variables were measured by Student’s *t* test. The Chi-square test was used to compare non-continues variables. Statistical significant was set at a p<0.05. Adverse or protective effects of FET on perinatal outcome versus fresh cycles are expressed as odds ratio.

## Results

1150 women using fresh ET and 300 women undergoing FET were initially enrolled the study. Sixteen women in fresh and 15 women in FET group were excluded because of refusing to participate or loss to follow up. Patents basic characteristics were not significantly different (Table I).

It is shown that FET cannot increase the chance of live birth (65.6% vs. 70.4% in fresh group) OR=0.80 (CI: 0.61-1.05) (p=0.120) (Table II). 79.1% of pregnancies in FET group were singleton, whereas 18.7% were twin and 2.2% were triple. It was observed that 69.4% of live births in fresh cycles lead to singleton pregnancies while twins and triples were 26.7% and 3.9% respectively (Table II). We found the comparable results regarding EP (0.7% vs. 0.9%), abortion (chemical and clinical pregnancy) (32.3% vs. 27.5%), and still birth (1.4% vs. 1.2%), in FET and fresh cycles respectively (Table II). 

The mean gestational age at the time of delivery was lower among singletons in FET group than fresh cycles (37.34±2.47 vs. 37.77±1.90 respectively) and the difference was statistically significant (p=0.047). Whereas, the mean gestational age of twin and triple pregnancies was significantly higher in FET group compared to fresh embryo transfer. There were no statistical differences in the mean live birth along with singleton and twin newborns between groups. Only triple pregnancies had a considerable higher birth weight in FET versus fresh cycles (p=0.028) (Table III). 

As it is presented in table IV, prematurity was significantly increased among singleton infants in FET group in comparison with neonates born after fresh ET (p=0.037). However, the percentage of premature twins was slightly elevated in fresh group. All of triple pregnancies in FET group and 90.3% in fresh cycles were premature. There was no statistically difference between two groups regarding SGA. Nevertheless, the proportion of LBW newborns were significantly decreased in twin and triple pregnancies in FET compared with fresh group. We observed the same sex ratio among singletons, twins and triples in FET group as well as fresh cycles (Table V).

**Table I:** Baseline characteristics of the patients in FET and Fresh cycle groups

**Variable**		**FET (n=285)**	**Fresh (n=1134)**	**p-value**
Maternal age (years)		30.53 ± 4.51	30.46 ± 4.84	0.907
Infertility duration* (years)		7 (IQR=5)	7 (IQR=6)	0.172
Causes of infertility				
	Male factor	126 (44.2%)	512 (45.1%)	0.915
	Ovary factor	64 (22.5%)	226 (19.9%)	
	Tubal factor	32 (11.2%)	129 (11.4%)	
	Endometriosis	3 (1.1%)	13 (1.1%)	
	Mixed	60 (21.1%)	254 (22.4%)	

Data are presented as mean ± SD.

FET: Frozen embryo transfer

* Median (Interquartile range).

**Table II:** Pregnancy outcome in FET women versus fresh cycle group

**Variable**	**FET**	**Fresh**	**Odds ratio (95% CI)**	**p-value**
Live birth	187 (65.6%)	798 (70.4%)	0.80 (0.61-1.05)	0.120
Singletons	148 (79.1%)	554 (69.4%)	1.67 (1.13-2.45)	0.009
Twins	35 (18.7%)	213 (26.7%)	0.63 (0.42-0.94)	0.025
Triples	4 (2.2%)	31 (3.9%)	0.54 (0.18-1.55)	0.253
Ectopic pregnancy	2 (0.7%)	10 (0.9%)	0.79 (0.17-3.64)	0.767
Pregnancy loss	92 (32.3%)	312 (27.5%)	1.25 (0.94-1.66)	0.111
Still birth	4 (1.4)	14 (1.2)	1.13 (0.37-3.48)	0.820

Data are presented as n (%).

FET: Frozen embryo transfer

**Table III:** Mean and standard deviation of gestational age and birth weight of two groups

**Variable**	**FET**		**Fresh**		**p-value**
	**Number**	**Mean±SD**	**Number**	**Mean±SD**	
Gestational age	187	37.04 ± 2.40	798	36.75 ± 2.83	0.190
Singletons	148	37.34 ± 2.47	554	37.77 ± 1.90	0.047
Twins	35	35.94 ± 1.78	213	34.78 ± 2.91	0.002
Triples	4	35.75 ± 0.50	31	31.90 ± 3.98	0.000
Birth weight	187	2798.40 ± 661.34	798	2687.55 ± 753.91	0.046
Singletons	148	2902.43 ± 663.58	554	2915.96 ± 664.53	0.826
Twins	35	2389.71 ± 483.48	213	2243.66 ± 648.27	0.204
Triples	4	2525.00 ± 556.02	31	1655.48 ± 725.80	0.028

Data are presented as mean ± SD.

FET: Frozen embryo transfer

**Table IV:** Prematurity, LBW and SGA in FET women versus fresh cycle group

**Variable**	**FET**	**Fresh**	**Odds ratio (95% CI)**	**p-values**
Prematurity	56 (29.9%)	244 (30.6%)	0.97 (0.68-1.37)	0.866
Singletons	29 (19.6%)	71 (12.8%)	1.65 (1.03-2.66)	0.037
Twins	23 (65.7)	145 (68.1)	0.89 (0.42-1.91)	0.782
Triples	4 (100%)	28 (90.3%)	1.10 (0.98-1.24)	1.000
LBW	48 (25.7%)	259 (32.5%)	0.71 (0.50-1.03)	0.072
Singletons	33 (22.3%)	105 (19%)	1.22 (0.78-1.90)	0.364
Twins	14 (40%)	128 (60.1%)	0.44 (0.21-0.91)	0.029
Triples	1 (25%)	26 (83.9%)	0.06 (0.00-0.74)	0.028
SGA	31 (16.6%)	180 (22.6%)	0.68 (0.44-1.03)	0.074
Singletons	21 (14.2%)	90 (16.2%)	0.85 (0.51-1.42)	0.543
Twins	9 (25.7%)	77 (36.2%)	0.61 (0.27-1.37)	0.233
Triples	1 (25%)	13 (41.9%)	0.46 (0.04-4.95)	0.523

Data are presented as numbers (%).

FET: Frozen embryo transfer

LBW: Low birth weight

SGA: Small for gestational age

**Table V:** Sex ratio in FET women versus fresh cycle group

**Variable**			**FET**	**Fresh**	**Odds ratio (95% CI)**	**p-values**
Sex						
	Boy		99 (52.9%)	415 (52%)	1.03 (0.75-1.42)	0.818
	Girl		88 (47.1%)	383 (48%)		
Singletons						
	Boy		81 (54.7)	299 (54)	1.03 (0.71-1.48)	0.869
	Girl		67 (45.3%)	255 (46%)		
Twins						
	Boy		16 (45.7%)	105 (49.3%)	0.86 (0.42-1.77)	0.695
	Girl		19 (54.3%)	108 (50.7%)		
Triples						
	Boy		2 (50%)	11 (35.5%)	1.81 (0.22-14.75)	0.576
	Girl		2 (50%)	20 (64.5%)		

Data are presented as numbers (%).

FET: Frozen embryo transfer

## Discussion

In this study, we compared perinatal outcomes after FET with fresh ET cycles. The main finding was that live birth slightly increased in fresh group without significant difference. As secondary outcomes, EP, spontaneous abortion and still birth did not differ between FET and fresh cycles. In our previous study, live birth regardless singletons or multiples was significantly lower in FET group (14). However in another studies chemical and clinical pregnancy rate as well as live birth in both singleton and multiple pregnancies did not vary between FET and fresh groups (11, 17). An important factor about developing live birth after fresh cycles may be transfer of top quality embryos. As freezing and thawing procedures are harmful for embryos, it is expected that only 30-48% of embryos survive intact after cryopreservation (18). However a recent study indicated that 66% of cycles present top quality embryo morphology after thawing (12). This argument is required to confirm by a prospective study using embryos with the same quality in both fresh and FET cycles.

Similar to older reports, our data showed no difference between two groups regarding spontaneous abortion (13, 19, 20). However, we found a significant miscarriage rate between FET and fresh group in our previous study (14). Similarly, a higher rate of spontaneous abortion ≤12 weeks in frozen-thawed embryos and 14% greater risk of miscarriage in thawed blastocyst transferred were reported compared to fresh ET (21, 22). Regarding ectopic pregnancy, there was no significant difference between FET and fresh cycles in current and our earlier research. Likewise, Levi *et al* and Jun found no significant alteration in EP between FET and fresh cycles (21, 23). Nevertheless, two other studies demonstrated that risk of EP significantly decrease by transfer of frozen blastocysts compared with fresh ones (24, 25). Similar to another study, the hazard of still birth did not differ significantly between two groups (10).

In contrast to other publications our data revealed that FET significantly increase the risk of prematurity and LBW in singleton pregnancies (8, 10, 26, 27). In our earlier study, prematurity and LBW were comparable between groups (14). Nevertheless, a meta-analysis on observational studies comparing perinatal outcome of FET and fresh cycles, confirmed that FET reduce the risk of LBW and prematurity in singletons (9). Conversely, another research demonstrated that LBW and prematurity have not any significant difference between fresh and FET groups among singleton and multiple pregnancies (11, 26). Shi et al also found that babies delivered after FET was significantly heavier than those born after fresh cycles in both singleton and multiple pregnancies (11). 

Another survey indicated that prematurity and LBW are 1.3 times and 1.5 times more common respectively in single tone pregnancies after fresh embryo transfer compared with FET. It is also showed that prematurity increased among couples with female factor infertility compared to male factors. However, in twins, preterm birth and LBW were decreased in ICSI and FET cycles and among couples with male factor infertility (28). It is showed in animal studies that both induction of ovulation and type of embryo culture medium disturb genomic imprinting and affect fetal outcome (29, 30). Recently, it is reported in human that the type of culture medium is significantly related to birth weight (31).

Otherwise, another study did not find any significant difference between two compared culture medium regarding mean birth weight, but babies born after cryopreservation had a significant higher birth weight than fresh group (32, 33). The authors believed that this alteration can be due to the interaction between cryo-protectants with the main enzyme interfered in epigenetic reprogramming, lead to normalization of the imprinting process (34). In accordance with some studies, we found that singleton and multiple pregnancies subsequent of FET showed a lower percentage of SGA newborns, but the difference was not statistically significant (9, 10, 26). 

In current study we did not find any difference in sex ratio (male/female) between fresh cycle and FET group. In our previous survey only singleton pregnancies showed a significant higher sex ratio in FET group compare to fresh embryo transfer (14). Similar to our finding, Wennerholm *et al* could not show any statistical significant difference in the sex ratio between singleton born after FET, fresh IVF/ICSI and spontaneous conception (10).

## Conclusion

In conclusion, according to our results, it seems that there is no major difference regarding perinatal outcome between fresh and frozen embryo transfer. Our data revealed that live birth did not differ significantly between FET and fresh cycles with a slight elevation in fresh group. The other outcomes including EP, spontaneous abortion (chemical and clinical pregnancy), still birth and SGA were similar in both groups. Our findings showed that FET significantly increases the risk of prematurity and LBW in singleton pregnancies.

## References

[1] Roque M, Lattes K, Serra S, Sola I, Geber S, Carreras R (2013). Fresh embryo transfer versus frozen embryo transfer in in vitro fertilization cycles: a systematic review and meta-analysis. Fertil Steril.

[2] Shapiro BS, Daneshmand ST, Garner FC, Aguirre M, Hudson C, Thomas S (2011). Evidence of impaired endometrial receptivity after ovarian stimulation for in vitro fertilization: a prospective randomized trial comparing fresh and frozen-thawed embryo transfer in normal responders. Fertil Steril.

[3] Shapiro BS, Daneshmand ST, Garner FC, Aguirre M, Hudson C, Thomas S (2011). Evidence of impaired endometrial receptivity after ovarian stimulation for in vitro fertilization: a prospective randomized trial comparing fresh and frozen-thawed embryo transfers in high responders. Fertil Steril.

[4] Devroey P, Bourgain C, Macklon NS, Fauser BC (2004). Reproductive biology and IVF: ovarian stimulation and endometrial receptivity. Trends Endocrinol Metab.

[5] Bourgain C, Devroey P (2003). The endometrium in stimulated cycles for IVF. Hum Reprod Update.

[6] Richter KS, Shipley SK, McVearry I, Tucker MJ, Widra EA (2006). Cryopreserved embryo transfers suggest that endometrial receptivity may contribute to reduced success rates of later developing embryos. Fertil Steril.

[7] Nayeri F, AghahosseiniAM, Alyasin A, Nili F (2006). Outcome of newborns conceived through artificial reproductive techniques in Tehran Iran. Iran JReprod Med.

[8] Pinborg A, Loft A, Aaris Henningsen AK, Rasmussen S, Andersen AN (2010). Infant outcome of 957 singletons born after frozen embryo replacement: the Danish National Cohort Study 1995-2006. Fertil Steril.

[9] Maheshwari A, Pandey S, Shetty A, Hamilton M, Bhattacharya S (2012). Obstetric and perinatal outcomes in singleton pregnancies resulting from the transfer of frozen thawed versus fresh embryos generated through in vitro fertilization treatment: a systematic review and meta-analysis. Fertil Steril.

[10] Wennerholm UB, Henningsen AK, Romundstad LB, Bergh C, Pinborg A, Skjaerven R (2013). Perinatal outcomes of children born after frozen-thawed embryo transfer: a Nordic cohort study from the CoNARTaS group. Hum Reprod.

[11] Shi W, Xue X, Zhang S, Zhao W, Liu S, Zhou H (2012). Perinatal and neonatal outcomes of 494 babies delivered from 972 vitrified embryo transfers. Fertil Steril.

[12] Veleva Z, Orava M, Nuojua-Huttunen S, Tapanainen JS, Martikainen H (2013). Factors affecting the outcome of frozen-thawed embryo transfer. Hum Reprod.

[13] Belva F HS, Van den Abbeel E, Camus M, Devroey P, Van der Elst J, Liebaers I (2008). Neonatal outcome of 937 children born after transfer of cryopreserved embryos obtained by ICSI and IVF and comparison with outcome data of fresh ICSI and IVF cycles. Hum Reprod.

[14] Aflatoonian A, Mansoori Moghaddam F, Mashayekhy M, Mohamadian F (2010). Comparison of early pregnancy and neonatal outcomes after frozen and fresh embryo transfer in ART cycles. J Assist Reprod Genet.

[15] Aflatoonian A, Yousefnejad F, Eftekhar M, Mohammadian F (2012). Efficacy of low-dose hCG in late follicular phase in controlled ovarian stimulation using GnRH agonist protocol. Arch Gynecol Obstet.

[16] Eftekhar M, Aflatoonian A, Mohammadian F, Eftekhar T (2013). Adjuvant growth hormone therapy in antagonist protocol in poor responders undergoing assisted reproductive technology. Arch Gynecol Obstet.

[17] Basirat Z, Adib Rad H, Esmailzadeh S, Jorsaraei SGA, Hajian- Tilaki K, Pasha H (2016). Comparison of pregnancy rate between fresh embryo transfers and frozen-thawed embryo transfers following ICSI treatment. Iran JReprod Med.

[18] Solé M SJ, Rodríguez I, Boada M, Coroleu B, Barri PN, Veiga A (2011). Correlation between embryological factors and pregnancy rate: development of an embryo score in a cryopreservation programme. J Assist Reprod Genet.

[19] Aytoz A VdAE, Bonduelle M, Camus M, Joris H, Van Steirteghem A, Devroey P (1999). Obstetric outcome of pregnancies after the transfer of cryopreserved and fresh embryos obtained by conventional in-vitro fertilization and intracytoplasmic sperm injection. Hum Reprod.

[20] Shen C, Shu D, Zhao X, Gao Y (2014). Comparison of clinical outcomes between fresh embryo transfers and frozen-thawed embryo transfers. Iran JReprod Med.

[21] Levi Setti PE, Albani E, Morenghi E, Morreale G, Delle Piane L, Scaravelli G, Patrizio P (2013). Comparative analysis of fetal and neonatal outcomes of pregnancies from fresh and cryopreserved/thawed oocytes in the same group of patients injection. Fertil Steril.

[22] Wang YA, Costello M, Chapman M, Black D, Sullivan EA (2011). Transfers of fresh blastocysts and blastocysts cultured from thawed cleavage embryos are associated with fewer miscarriages. Reprod Biomed Online.

[23] Jun SH MA (2007). Ectopic pregnancy rates with frozen compared with fresh blastocyst transfer. Fertil Steril.

[24] Li Z, Sullivan EA, Chapman M, Farquhar C, Wang YA (2015). Risk of ectopic pregnancy lowest with transfer of single frozen blastocyst. Hum Reprod.

[25] Ishihara O, Kuwahara A, Saitoh H (2011). Frozen-thawed blastocyst transfer reduces ectopic pregnancy risk: an analysis of single embryo transfer cycles in Japan. Fertil Steril.

[26] Pelkonen S, Koivunen R, Gissler M, Nuojua-Huttunen S, Suikkari AM, Hyden-Granskog C (2010). Perinatal outcome of children born after frozen and fresh embryo transfer: the Finnish cohort study 1995-2006. Hum Reprod.

[27] Kallen B, Finnstrom O, Nygren KG, Olausson PO (2005). In vitro fertilization (IVF) in Sweden: infant outcome after different IVF fertilization methods. Fertil Steril.

[28] Wang YA, Sullivan EA, Black D, Dean J, Bryant J, Chapman M (2005). Preterm birth and low birth weight after assisted reproductive technology-related pregnancy in Australia between 1996 and 2000. Fertil Steril.

[29] Market-Velker BA, Fernandes AD, Mann MR (2010). Side-by-side comparison of five commercial media systems in a mouse model: suboptimal in vitro culture interferes with imprint maintenance. Biol Reprod.

[30] Dumoulin JC, Land JA, Van Montfoort AP, Nelissen EC, Coonen E, Derhaag JG (2010). Effect of in vitro culture of human embryos on birthweight of newborns. Hum Reprod.

[31] Nelissen EC, Van Montfoort AP, Coonen E, Derhaag JG, Geraedts JP, Smits LJ (2012). Further evidence that culture media affect perinatal outcome: findings after transfer of fresh and cryopreserved embryos. Hum Reprod.

[32] Eaton JL, Lieberman ES, Stearns C, Chinchilla M, Racowsky C (2012). Embryo culture media and neonatal birthweight following IVF. Hum Reprod.

[33] Vergouw CG, Kostelijk EH, Doejaaren E, Hompes PG, Lambalk CB, Schats R (2012). The influence of the type of embryo culture medium on neonatal birthweight after single embryo transfer in IVF. Hum Reprod.

[34] De Geyter C, De Geyter M, Steimann S, Zhang H, Holzgreve W (2006). Comparative birth weights of singletons born after assisted reproduction and natural conception in previously infertile women. Hum Reprod.

